# Health care costs attributable to overweight calculated in a standardized way for three European countries

**DOI:** 10.1007/s10198-014-0655-8

**Published:** 2014-11-29

**Authors:** M. Lette, W. J. E. Bemelmans, J. Breda, L. C. J. Slobbe, J. Dias, H. C. Boshuizen

**Affiliations:** 1 National Institute for Public Health and the Environment, Centre for Nutrition, Prevention and Health Services, P.O. Box 1, 3720 BA Bilthoven, The Netherlands; 2 WHO Regional Office for Europe, Nutrition, Physical Activity and Obesity, Marmorvej 51, 2100 Copenhagen, Denmark; 3 Department of Clinical Sciences in Malmö, Lund University, Clinical Research Center 60:13:36, Jan Waldenströms Gata 35, 20502 Malmö, Sweden

**Keywords:** Overweight, Cost calculation, Health care costs, Macrolevel data, C82, I19

## Abstract

This article presents a tool to calculate health care costs attributable to overweight in a comparable and standardized way. The purpose is to describe the methodological principles of the tool and to put it into use by calculating and comparing the costs attributable to overweight for The Netherlands, Germany and Czech Republic. The tool uses a top-down and prevalence-based approach, consisting of five steps. Step one identifies overweight-related diseases and age- and gender-specific relative risks. Included diseases are ischemic heart disease, stroke, hypertension, type 2 diabetes mellitus, colorectal cancer, postmenopausal breast cancer, endometrial cancer, kidney cancer and osteoarthritis. Step two consists of collecting data on the age- and gender-specific prevalence of these diseases. Step three uses the population-attributable prevalence to determine the part of the prevalence of these diseases that is attributable to overweight. Step four calculates the health care costs associated with these diseases. Step five calculates the costs of these diseases that are attributable to overweight. Overweight is responsible for 20–26 % of the direct costs of included diseases, with sensitivity analyses varying this percentage between 15–31 %. Percentage of costs attributable to obesity and preobesity is about the same. Diseases with the highest percentage of costs due to overweight are diabetes, endometrial cancer and osteoarthritis. Disease costs attributable to overweight as a percentage of total health care expenditures range from 2 to 4 %. Data are consistent for all three countries, resulting in roughly a quarter of costs of included diseases being attributable to overweight.

**Electronic supplementary material:**

The online version of this article (doi:10.1007/s10198-014-0655-8) contains supplementary material, which is available to authorized users.

## Introduction

The prevalence of overweight (BMI ≥ 25 kg/m^2^) and obesity (BMI ≥ 30 kg/m^2^) is rapidly increasing in the WHO European Region. In 2008/2009, the prevalence of overweight in 19 European Union member states varied between 51 and 69 % for men and between 37 and 57 % for women [1]. If recent trends continue unabated, in 2030 there will be 2.16 billion overweight and 1.12 billion obese individuals worldwide [2]. Obesity is one of the WHO Regional Office for Europe’s top priorities and the ministerial conference on nutrition in November 2006 in Istanbul therefore completely focused on obesity [3].

Overweight is associated with increased risks for several chronic diseases, especially type 2 diabetes mellitus, cardiovascular diseases and musculoskeletal disorders [4]. Obesity at age 40 has been shown to reduce life expectancy by 7 years in women and 6 years in men [5]. The increased prevalence of chronic diseases that are partially due to overweight causes a large burden on the health care system and is associated with considerable health care costs. Quantification of the amount of health care costs attributable to overweight contributes to increased political awareness to take action against it. Previous research in various countries showed that between 2 and 5 % of annual health care costs are attributable to overweight [6–11]. However, several reviews on the cost of illness attributable to overweight show that, due to different methods for calculation of these costs, the results are often not mutually comparable between countries [10, 12, 13]. Furthermore, several of these methods require data that are not generally available. Therefore, the WHO regional office for Europe commissioned the Dutch National Institute for Public Health and the Environment (RIVM) to develop a methodology for estimating the costs attributable to overweight in a standardized way and to implement this in a software tool (the OBCOST tool). The methodology should make use of data that are generally available for most countries.

The purpose of this article is to describe this software tool and methodological principles behind it. Furthermore, the tool is put into use by calculating and comparing the costs attributable to overweight among three European countries, i.e., The Netherlands (NL), Germany (GE) and Czech Republic (CZR).

## Methods and procedures

### Methodology of the OBCOST tool

**Fig. 1:**
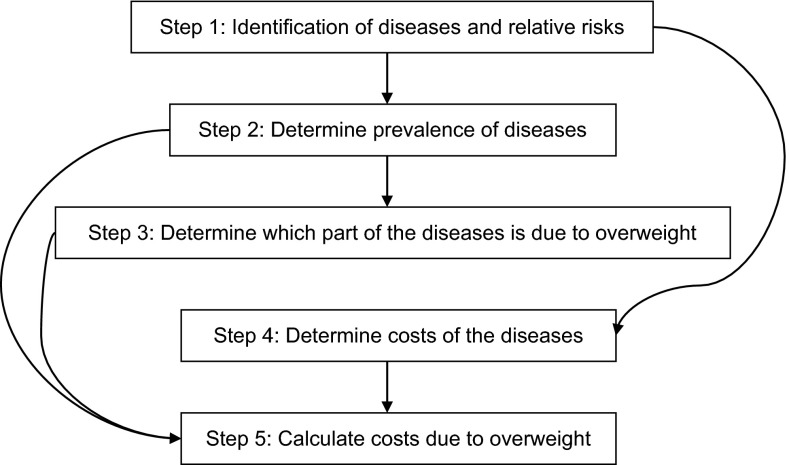
General framework of the OBCOST methodology

The OBCOST tool uses a top-down, prevalence-based approach, answering the question: what current (annual) health care costs would have been avoided if overweight had been eliminated in the past? The general framework of the tool consists of five steps, which are shown in Fig. 1. Below, each step will be described shortly. Background information on the methodology and OBCOST tool can be found in Online Resource 1.

In step one, diseases are identified that are related to overweight, and age- and gender-specific relative risks are assessed. For pragmatic reasons, this study uses the WHO Comparative Quantification of Health Risks [4] where the following diseases are estimated to be associated with overweight: ischemic heart disease (IHD) (ICD-10: I20–I25), stroke (ICD-10: I60–I69), hypertensive disease (ICD-10: I10–I13), type II diabetes mellitus (ICD-10: E11), colorectal cancer (ICD-10: C18–C21), postmenopausal breast cancer (ICD-10: C50), endometrial cancer (ICD-10: C54–C55), kidney cancer (ICD-10: C64–68) and osteoarthritis (ICD-10: M15–M19). The age- and gender-specific relative risks that were entered in the tool consist partly of relative risks given by the WHO in their Comparative Quantification of Health Risks and partly of relative risks as used by the RIVM in their chronic disease modelling (see Online Resource 2).

Step two consists of collecting data on the age- and gender-specific prevalence of these diseases [*p*_d_(a, g)]. These *p*_d_(a, g) data are assumed to be available from surveys or morbidity registers, or they can be calculated (by the OBCOST tool) from incidence data and disease-specific mortality using an incidence-prevalence-mortality (IPM) model [14]. The latter is especially relevant for diseases, such as cancers, where incidence data are more readily available than prevalence data. Furthermore, in this step collection of prevalence data for preobesity (BMI 25 < 30 kg/m^2^) and obesity (BMI > 30 kg/m^2^) is required.

The third step determines the part of the prevalence of the included diseases that is attributable to overweight. The population-attributable prevalence (PAP) is used instead of the population-attributable risk (PAR) since for chronic diseases health care costs are (mostly) related to the number of prevalent cases in a population and not to the number of incident cases. Therefore, prevalence rates will provide a more comprehensive estimation of costs than incidence rates. Furthermore, the PAP takes into account that risk factors and their relative risks can change over time, as opposed to the PAR. The part of a disease that can be attributed to overweight can be defined using the following formula:

$${\text{PAP}}_{\text{d}} ( {\text{a, g)}} = \frac{{p_{\text{d}} ( {\text{a, g)}}\,\text{ - } \, \it p_{\text{d}} ( {\text{a, g|overweight}} \to {\text{ normal)}}}}{{p_{\text{d}} ( {\text{a, g)}}}}$$

where *p*_d_(a, g) is the prevalence of disease d for age group a and gender g and *p*_d_(a, g |overweight → normal) is the prevalence of disease d for age group a and gender g in a situation when all overweight persons would have had a normal weight. The PAP is determined by back calculation of past incidence rates from prevalence rates and disease-specific mortality rates using the IPM (incidence-prevalence-mortality) model [14]. Then, relative risks are used to calculate what the past incidence rates would have been in a population without overweight. These past incidence rates are calculated back to current prevalence rates in a hypothetical population without overweight, and then the PAP is found from the difference between these hypothetical prevalence rates and the observed prevalence rates. For a more detailed description of this method, see Online Resource 1.

Step four calculates the health care costs associated with the included diseases [c_d_(a, g)]. The method uses a top-down approach for estimating the cost of illness, which consists of four stages [15, 16]. First, total health care costs of different health care providers are calculated. Second, information on health care utilization is collected for each health care provider from patient registers or surveys. These data should contain information on health care use by the included diseases for each provider, and if possible, information should be stratified by age and gender. Third, allocation keys need to be identified for each health care provider. These allocation keys define the resource use by each disease. In the last stage, all information is combined, where total costs per provider are allocated to diseases. The cost of illness for all providers is summed up, which results in the final cost of illness estimation for each disease by age and gender, c_d_(a, g). In order to assure international comparability of cost of illness estimations, the use of a standardized health accounting framework is required. The OECD System of Health Accounts (SHA) is recommended, restricting costs to curative care [17].

Finally, the fifth step calculates the costs attributable to overweight with the following formula:

$$\rm Cost = \sum\limits_{a} {\sum\limits_{g} {\sum\limits_{d} {c_{\rm {d}} (a, g)p_{\rm{d}} (a, g){\rm PAP_{\rm{d}} }} } } (a, g)$$

using the appropriate PAP_d_ value for each disease. The tool provides results in both absolute costs of included diseases that are attributable to overweight and percentage of costs of included diseases that are attributable to overweight.

### Data collection for the three countries and data treatment

In order to perform calculations for NL, GE and CZR, data from these three countries were collected for population numbers, BMI prevalence, mortality of diseases, disease prevalence/incidence and costs of diseases. If possible, collected data were age and gender specific (5-year age groups were used). Data were not always complete for all age categories. Since the OBCOST software does not work when one or more cells are incomplete, estimations were made for missing data. Detailed information on the collected data and assumptions made is provided in Online Resource 3.

The most important assumptions are presented here in short. First, GE reported their population numbers of the eldest ages as an aggregate group (90+). This number was divided over the last three original age categories (90–94, 95–99, 100+) according to the WHO world standard population distribution [18]. Second, for all countries, prevalence, mortality and cost data were sometimes reported in aggregate age groups as well. For prevalence and mortality data, it was assumed that the rate provided for the aggregate age group could be applied to all original age groups within this aggregate age group. For cost data, the number provided for the aggregate age group was divided by the number of original age groups within the aggregate age group, and this result was applied to all original age groups within this aggregate age group. Lastly, since the OBCOST tool uses the IPM model to calculate incidence from prevalence and mortality data, prevalence data have to be sufficiently smooth in order to prevent generating negative incidences. When prevalence data were not increasing monotonously, these data had to be smoothed in order for the OBCOST software to work properly. Slight deviations in the smoothness of the prevalence data were fixed by averaging the prevalence rate causing the tool to err with the prevalence rate in the previous age group, and using this average for both age groups.

### Sensitivity analyses

Sensitivity analyses were performed in several ways. First, the sensitivity of the tool for missing disease data was estimated by including estimations of these missing disease data based on the data in the other countries in sensitivity calculations. The missing diseases categories were estimated by averaging data for the missing disease categories from the other two countries. Second, sensitivity of the tool to changes in disease costs was estimated by including pharmaceutical costs for hypertension and diabetes in cost data for CZR. These pharmaceutical costs were not included in the cost of illness for CZR, but were provided separately based on the Anatomical Therapeutic Chemical (ATC) classification system. Third, sensitivity analyses were performed with both of the previously described situations at the same time. Fourth, the sensitivity of the tool to variations in BMI prevalence was estimated, since studies have shown that self-reported BMI tends to differ from measured BMI by overestimating BMI at the lower end of the BMI scale (BMI < 22) and underestimating BMI at the higher end of the BMI scale (BMI > 28) [19, 20]. The effect of these variations was estimated by increasing BMI prevalence with 0.56 for preobesity and 1.16 for obesity [19, 20]. Lastly, the effect of exclusion of different types of disease categories was estimated by excluding various diseases one by one from all analyses.

## Results

**Fig. 2:**
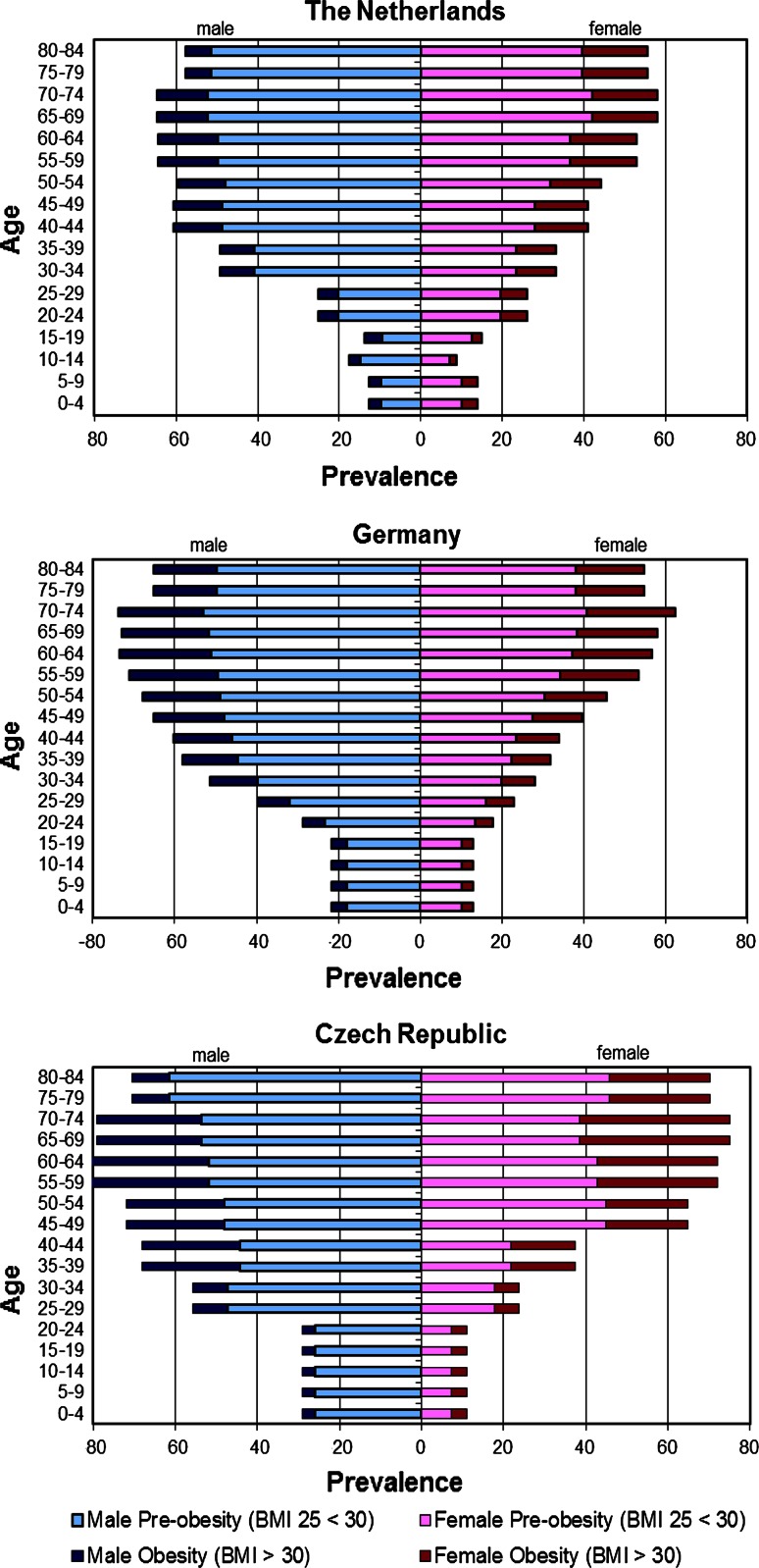
BMI distributions across age for each country

As could be expected, the proportion of males (49 %) and females (51 %) in the population is the same for all three countries. Table 1 shows population data and summarized disease data for each country. The proportion of the population older than 55 years ranges from 28 % for NL to 33 % for GE. As can be seen in Fig. 2, which presents the BMI distribution across age categories for the three countries, the prevalence of overweight increases from age 20 to age 74. The absolute prevalence of overweight is highest between the ages of 55 and 74. In NL, this prevalence ranges from approximately 50 % for females to about 60 % for males, while in GE it rises to approximately 60 % for females and 70 % for males. In CZR the prevalence of overweight is highest, with almost 80 % for both sexes.

**Table 1:** Country data: population composition and summarized disease prevalence/incidence

	The Netherlands	Germany	Czech Republic
Population composition			
Total population (age 0–100+)	16.574.989	81.757.471	10.517.247
Population aged >55 (%)	28	33	30
Disease prevalence/incidence (per 100 person years) in total population^a^			
IHD (%)	10^b^	10^b^	9^b^
Stroke (%)	3^b^	5^b^	4^b^
Hypertension (%)	21^b^	29^b^	30^b^
Diabetes (%)	7^b^	6^b^	17^b^
Colon cancer (%)	0.15^c^	1^b^	0.15^c^
Breast cancer (%)	0.32^c^	1^b^	0.08^c^
Endometrial cancer (%)	0.02^c^	0.02^c^	0.02^c^
Kidney cancer (%)	0.07^c^	0.08^c^	0.12^c^
Osteoarthritis (%)	9^b^	20^b^	10^b^

^a^Summarized disease data are presented as provided by the different countries and entered into the OBCOST tool in order to show the flexibility of the tool in handling the differences between different countries in collecting and registering data (e.g., the use of prevalence or incidence rates for certain diseases)

^b^Disease prevalence

^c^Disease incidence (per 100 person years)

**Fig. 3:**
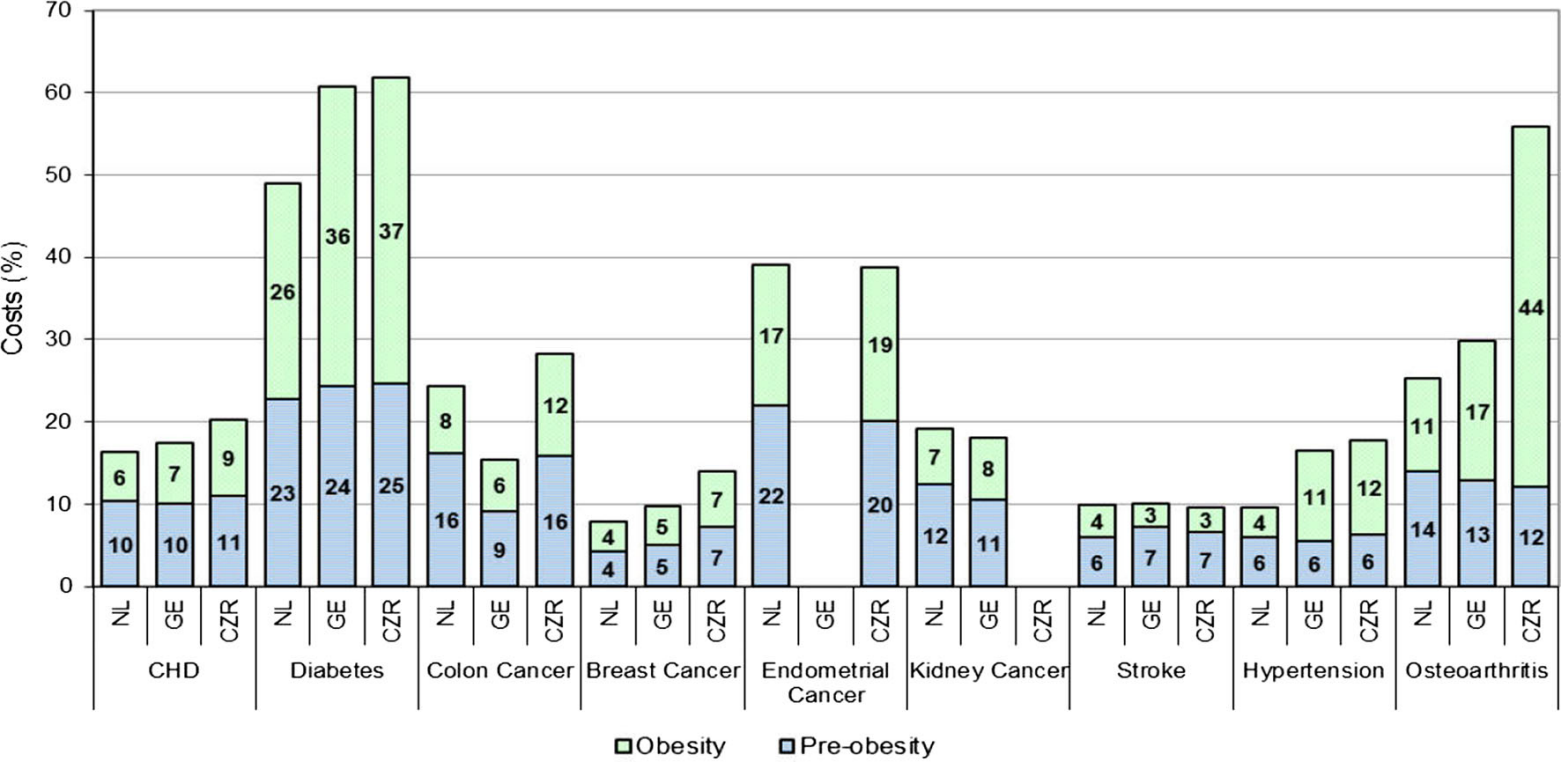
Percentage of costs attributable to overweight for each disease, with preobesity and obesity presented separately

The summarized disease data in Table 1 do not show major differences between countries except for diabetes prevalence in CZR and osteoarthritis prevalence in GE, which are both much higher than in the other countries. Table 2 presents annual costs for all included diseases for each country together with the (percentages of) costs that can be attributed to preobesity, obesity or overweight in total. Despite large differences in total annual costs and costs per capita, the results show that between one-fifth and a quarter of total disease costs can be attributed to overweight, with percentages ranging from 20 to 26 %. Furthermore, the percentage of costs attributable to obesity and preobesity is about the same. Figure 3 shows the percentages of costs that can be attributed to preobesity and obesity for each disease separately, diabetes, endometrial cancer and osteoarthritis being the three leading diseases with the highest percentages of respectively about 50–60 %, about 38 % and about 25–55 %. In terms of absolute attributable costs, the diseases with the highest costs attributable to overweight are diabetes (€444 million, €3.6 billion and €60 million for NL, GE and CZR respectively), IHD (€267 million, €1 billion and €43 million for NL, GE and CZR respectively) and osteoarthritis (€142 million, €2 billion and €24 million for NL, GE and CZR respectively).

**Table 2:** Absolute costs and percentage of costs of included diseases attributable to overweight

	Total costs in €	Costs per capita in €^a^	Costs due to preobesity in € (% of total costs)	Costs due to obesity in € (% of total costs)	Costs due to overweight in € (% of total costs)
The Netherlands	6.029.469.861	476	657.302.148 (11)	528.386.496 (9)	1.185.688.644 (20)
Germany	38.737.666.667^b^	582	4.232.431.091 (11)	5.154.965.842 (13)	9.387.396.934 (24)
Czech Republic	763.339.408^c^	91	90.612.458 (12)	108.310.987 (14)	198.923.446 (26)

^a^For the population aged 20+

^b^Excluding endometrial cancer

^c^Excluding kidney cancer and pharmaceutical costs

The percentages of costs attributable to overweight calculated here are percentages of total costs of included diseases, as opposed to percentages of total annual health care expenditures. Estimation of the percentage of total health care costs that are attributable to overweight (based on the costs of diseases included in this analysis) results in percentages of 2.3 % for NL, 2.1 % for CZR and 3.7 % for GE.

### Sensitivity analyses

Table 3 shows the effects of various sensitivity analyses compared with the original model. In the first analysis, the missing disease categories endometrial cancer and kidney cancer for GE and CZR respectively are added to the original model, which does not result in any changes. However, the model is sensitive to variations in cost data. Inclusion of pharmaceutical costs for diabetes and hypertension for CZR results in an increase of the percentage of total costs attributable to overweight from 26.1 to 30.6 %. Inclusion of both the estimations for kidney cancer and these pharmaceutical costs lowers the percentage to 29.7 % for CZR. Increases in BMI prevalence as described in the fourth sensitivity analysis resulted in an increase of the percentage of total disease costs attributable to overweight of 0.7 % for NL and GE and of 0.6 % for CZR. Exclusion of diseases as described in the last sensitivity analysis has various effects, depending on the type of disease. Exclusion of endometrial and kidney cancer does not result in consequential changes of the percentage of costs attributable to overweight. Exclusion of stroke increases the percentage of total costs attributable to overweight with 2.7 % for NL, 3.1 % for GE and 3.6 % for CZR. Exclusion of diabetes lowers the percentage of total costs that are attributable to overweight with 5.2 % for NL, 6.5 % for GE and 5.3 % for CZR.

**Table 3:** Changes in percentage of total disease costs attributable to overweight after sensitivity analyses

	The Netherlands	Germany	Czech Republic
Original (%)	19.7	24.2	26.1
All diseases equal (%)	19.7	24.3^a^	25.5^b^
All cost categories equal (%)	19.7	24.2	30.6^c^
Both equal (%)	19.7	24.3	29.7
Adjusted BMI (%)	20.4	24.9	26.7
Excluding various diseases (%)			
Endometrial cancer	19.7	–	25.8
Kidney cancer	19.7	24.4	–
Osteoarthritis	19.1	23.2	24.2
Diabetes	14.5	17.7	20.8
Stroke	22.4	27.3	29.7

^a^Costs for endometrial cancer estimated based on costs from NL and CZR

^b^Costs for kidney cancer estimated based on costs from NL and GE

^c^Pharmaceutical costs for hypertension and diabetes included

## Discussion

The estimated percentage of total costs of nine obesity-related diseases attributable to overweight ranges from 20 to 26 % in this analysis. Main contributors to this high percentage are diabetes, endometrial cancer and osteoarthritis. The present results are based on data provided by different countries with their own ways of collecting and registering data. This has some consequences for the quality and comparability of the data entered into the tool.

Data for the eldest age groups were often not available. Estimations were made by applying the rate of the last available age group to all successive age groups. Since the eldest age groups (85+) are very specific groups, these assumptions are very precarious and can possibly bias the results. Due to the assumptions made, exclusion of these eldest age groups from this analysis had little effect on the percentage of total disease costs attributable to overweight. In general, the eldest age groups have a larger disease burden compared with the younger age groups. However, much of this disease burden is due to non-overweight-related diseases, so a relatively smaller share of the disease burden in the eldest age groups is attributable to overweight. Therefore, the percentage of disease costs attributable to overweight in the eldest age groups can be expected to be relatively low and pull down the percentage of total disease costs attributable to overweight. However, due to the small size and relatively low absolute costs of the eldest age groups (regarding the diseases included in this analysis), this influence might not be significant.

Due to variations and uncertainties regarding the included diseases, cost data and BMI data, various sensitivity analyses were performed that yielded only small effects. Since including estimations of missing data did not change the percentages of total disease costs attributable to overweight, slight deviations in the ICD-10 codes for which disease data were provided can be expected to have negligible effects as well. Inclusion of pharmaceutical costs for CZR did result in a percentage change, suggesting that differences in cost data can influence cost estimates. Cost data are preferably based on the functional classification of the SHA [17] in order to ensure maximal comparability. However, many countries differ in their level of implementation of this classification [15, 21], which can result in considerable bias in comparisons. Variations in self-reported and measured BMI data are unlikely to bias comparisons between countries, since sensitivity analyses showed only a small effect on the percentage of total disease costs attributable to overweight.

This study uses a method in which current disease prevalence data are used to estimate past incidence rates, which in turn are used to calculate current prevalence rates in a hypothetical population without overweight. However, since BMI prevalence rates have changed over time [22], the method should also apply past BMI prevalence rates to past incidence rates in order to be completely accurate. However, in order to do this, more complex methods are needed. This undermines the aim of this tool. However, it should be kept in mind that since BMI prevalence rates may change with different dynamics in different countries [23], this might affect the comparability of the results.

Some methodological issues are discussed according to their potential influence as opposed to previous calculations. First, the results of this method will depend on the number and type of diseases that are included in the calculations; this will influence both absolute costs and percentage of costs attributable to overweight. Generally, when more diseases are included, estimated absolute costs attributable to overweight will increase. The estimated percentage of costs attributable to overweight is strongly dependent on the PAP of the included diseases. When diseases have a low PAP (such as stroke), a relatively small share of the costs of these diseases will be attributable to overweight. Therefore, these diseases will lower the percentage of total costs of included diseases attributable to overweight. On the other hand, diseases with a high PAP (such as diabetes) will result in an increase of this percentage.

Second, the percentages of costs attributable to overweight calculated by the OBCOST tool are percentages of total costs of included diseases, as opposed to percentages of total annual health care expenditures. Estimation of the percentage of total health care costs that are attributable to overweight (based on the costs of diseases included in this analysis) results in percentages of 2.3 % for NL, 2.1 % for CZR and 37 % for GE. This is in line with results found in previous studies, where percentages were found ranging from 2.1 % of total health care costs in Germany to 4.8 % of total health care spending in the US [6–11].

Third, by using PAPs, the method assumes that overweight-related diseases are mutually exclusive. However, overweight people often have multiple attributable diseases at a time, and often interactions exist between these conditions. The dynamics of these interactions are not reflected in the relative risks used for the PAPs. When health care provision for multiple diseases becomes more efficient (for example, due to the implementation of the chronic care model [24]), this will result in an overestimation of the estimated costs attributable to overweight.

Furthermore, when interpreting the results of this study it should be kept in mind that the presented costs consist of direct (medical) costs attributable to overweight. They do not take into account indirect costs such as production losses due to morbidity, mortality or informal care. Inclusion of indirect costs can lead to much higher estimates of total costs attributable to overweight. A review by Trogdon et al. (2008) [25] found absolute indirect costs of obesity ranging between $448 million ($204 per obese person) in Switzerland and $66 billion ($1,627 per obese person) in the USA. In Canada, indirect costs attributable to overweight were estimated to be $5 billion, constituting 9.5 % of total indirect costs (total indirect costs in this study consisted of short- and long-term morbidity costs for 18 comorbidities, including the diseases included in this analysis) [9]. Estimations of indirect costs should be added up to the estimated direct medical costs in order to determine total costs attributable to overweight. The absolute costs presented in this study will therefore be an underestimation of the total costs attributable to overweight and obesity.

### Practical implications

Since the OBCOST tool uses data that are supposed to be generally available for many countries, the tool can be used by any country where BMI data are available and where health care expenditure data are available coded by disease. When this is not the case, estimations can be made by using foreign data. The standardized method of cost calculations will lead to more comparable estimates of costs of overweight between countries. However, due to the flexible nature of the tool, expansion of its functionality is supported should the user find this necessary. For instance, one can include the nonmedical costs of disease or extend the selection of diseases by adding other diseases known to be associated with overweight [26].

Information about costs attributable to overweight is important for establishing a case of preventive action [10]. By using a prevalence-based approach, this method is particularly suited for estimating the magnitude of the annual economic burden attributable to overweight. However, it does not provide information on the long-term consequences of overweight and the value of specific interventions that may lessen the burden of disease. To obtain this kind of information, an incidence-based approach is more appropriate, identifying what future lifetime costs would be avoided if all new overweight cases would be eliminated during a certain year. Successful prevention of overweight will lead to a decrease in the percentage of health care costs attributable to overweight, as a larger part of the health care costs will be due to age-related diseases and not overweight-related diseases. However, for the effect of successful prevention on total health care costs, also costs of life years gained need to be taken into account. Generally, successful prevention will result in an increase in life expectancy, in which people will suffer from other diseases. This will increase total health care costs in the long term. However, because overweight is related to some low-mortality but high-cost diseases such as osteoarthritis, the ratio of cost savings due to reduced incidence of risk factor-related diseases and the medical costs of life years gained is more favorable for overweight prevention than for example for smoking prevention [27]. These future costs, together with the costs of preventive interventions themselves, are relevant when cost calculations are performed in order to support health care decision makers in formulating specific health policies. However, if the aim is to use attributable costs as an indicator of conditions because of past policies, the prevalence-based approach is suitable. Furthermore, data needed for an incidence-based approach are more complex and often less readily available, which makes the prevalence-based approach more applicable.

This tool can be relevant for several purposes. In the first place, it can serve to increase knowledge as a long-term monitoring instrument in order to keep track of changes in the annual economic burden of overweight. Furthermore, the tool could also be developed for other risk factors of disease. This way, the tool can be used to monitor and compare between costs attributable to different risk factors. This kind of information could be helpful in determining which risk factors or diseases are most costly and how to distribute preventive resources.

## Conclusion

The present study described a standardized methodology for calculating health care cost attributable to overweight. This methodology increases comparability between countries. Results show that for the three countries included, roughly a quarter of the costs of included diseases are attributable to overweight. Information about the costs of overweight increases political awareness and emphasizes the need for preventive action.

## Electronic supplementary material

Online Resources provide background information on the OBCOST tool and its methodology (Online Resource 1), an overview of relative risks entered into the tool (Online Resource 2) and detailed information on collected data and assumptions made (Online Resource 3).

Below is the link to the electronic supplementary material.

Supplementary material 1 (DOCX 102 kb)

Supplementary material 2 (DOCX 37 kb)

Supplementary material 3 (DOCX 23 kb)
