# Supplementary material for: Health care costs attributable to overweight calculated in a standardized way for three European countries
Source: Eur J Health Econ. 2014 Nov 29;17(1):61–9. doi: 10.1007/s10198-014-0655-8 (PMC4705131; doi:10.1007/s10198-014-0655-8)
Supplement: Supplementary file 1 — Supplementary material 1 (DOCX 102 kb) [file 10198_2014_655_MOESM1_ESM.docx]

**Online Resource 1**

**Background information on methodology and OBCOST tool**

**Journal**

European Journal of Health Economics

# **Title**

# Health care costs attributable to overweight calculated in a standardized way for three European countries

# **Authors**

# M. Lette, W.J.E. Bemelmans, J. Breda, L.C.J. Slobbe, J. Dias, H.C. Boshuizen

#

# **Corresponding author**

# Manon Lette, MSc, National Institute for Public Health and the Environment, Centre for Nutrition, Prevention and Health Services, PO Box 1, 3720 BA Bilthoven, The Netherlands

# e-mail: [manon.lette@rivm.nl](mailto:manon.lette@rivm.nl)

**OBCOST: a tool to calculate health care costs attributable to overweight**

The general purpose of the OBCOST tool is to provide each country the possibility to estimate the health care costs attributable to overweight based on a minimal requirement of country specific data. Overall principles of the OBCOST tool are: 1) the tool estimates prevalence based costs, and 2) the method uses cost of illness data from a top-down cost of illness assessment.

**Prevalence based methodology**

With a prevalence-based methodology, the OBCOST tool calculates the annual costs of overweight. Costs of overweight are calculated by determining which part of the included diseases is attributable to overweight. To determine this, the population attributable prevalence (PAP) is used instead of the population attributable risk (PAR), since for chronic diseases health care costs are (mostly) related to the number of prevalent cases in a population, and not to the number of incident cases. This is a simplification of reality that might not always be fully justified. Some diseases yield high costs shortly after diagnosis, and much lower costs in the later, chronic stage. In other cases costs accrued shortly before death are dominant.

The PAP is defined as the difference between the real prevalence of disease d in the population, and the prevalence when all obese persons would have had a normal weight (expressed as a fraction of the real prevalence). To estimate the prevalence of disease in absence of obesity, results from epidemiological studies in the form of relative risks are used, together with information on the prevalence of obesity. Two problems need to be tackled with this estimation. First, relative risks from epidemiological studies apply to incidence rates and not to prevalence rates. In order to determine the proportion of prevalent cases attributable to overweight, the incidence history needs to be reconstructed. Second, the method should not compare the prevalence in the overweight population with the prevalence in the population with normal weight, but it should compare the prevalence in the overweight population with the prevalence that this population would have had when the overweight would have had normal weight. For instance, both overweight and diseases are more frequent in middle age than at younger ages. This has to be taken into account in this comparison: the population should not be rejuvenated by removing overweight.

Figure 1 gives the outline for the method to determine the PAP. By assuming a stationary population, it is possible to back calculate incidence rates from prevalence rates and disease specific mortality rates, using an Incidence-Prevalence-Mortality (IPM) model [[1](#_ENREF_1)]. This will be described in more detail in the next paragraph. Relative risks and population prevalence rates of overweight are then used to calculate how large the incidence rates would have been in a population without overweight. From these incidence rates, prevalence rates of disease in the hypothetical population without overweight are calculated, using the inverse procedure of the back-calculation (see next paragraph). The PAP is then found from the difference between the prevalence of the disease in the hypothetical overweight free population and the observed prevalence. With using the IPM model, it is important that prevalence data is monotonously increasing. When this is not the case and excess mortality rates are too low to compensate this, the model will generate negative incidences. In order to prevent this, prevalence data should be sufficiently smooth.

Fig. 1 Steps in calculating the Population Attributable Prevalence

**Calculating prevalence rates of disease in a hypothetical population without overweight**

The incidence history of the population is reconstructed using an IPM model (see figure 2). It is assumed that remission is absent. In the IPM model, mortality in those with a disease is split into: 1) usual mortality, that is mortality equal to that of persons (of similar age and gender) without the disease, and 2) excess mortality, that is mortality that exceeds the usual mortality, because of having the disease. The IPM model for independent diseases without remission is described by the following differential equation that determines the change of prevalence [[2](#_ENREF_2)]:

(eq. 1)

p_d_(a): prevalence rate of disease d at age a

i_d_(a): incidence of disease d at age a

E_d_(a): excess mortality of disease d at age a, which is the difference in mortality between those with and without disease d.

If we assume that the incidence i_d_(a) and excess mortality E_d_(a) are piecewise constant (that is i_d_(a1) and E_d_(a1) are constant between a=a1 up to but not including a=a2), this yields the following solution for p_d_(a2):

(eq. 2)

The prevalence rates p_d_(a2) and p_d_(a1) are known, as they are input to the model. Thus we only need excess mortality E_d_(a1) in order to find the incidence i_d_(a1) for each age from this formula (using numerical root finding methods). Excess mortality E_d_(a1) is calculated from cause specific mortality due to disease d at age a1 by dividing the cause specific mortality with the fraction of prevalent cases p_d_(a1).

The back calculated incidence rates are overall incidence rates, thus for all BMI groups taken together. The next step is to separate this overall incidence into separate incidences for those who are obese, have overweight or normal weight. For this, we use the epidemiological relationship:

(eq. 3)

where RR_obese,d_ is the relative risk for obesity on incidence of disease d, and i_normal,d_ is the incidence of disease d in those with normal weight, leaving out the dependence on age to simplify the notation. A similar formula can be written for overweight. The incidence in the entire population is a weighted mean of the incidence in those with normal weight, overweight and obesity:

(eq. 4)

Here, i_d_ has been obtained from the back calculation, while the RRs and prevalence rates p_d_ are input to the method. This means that we can use eq. 4 to find i_normal,d_.

The last step of our procedure calculates the prevalence in those without obesity by starting at age zero, assuming a zero prevalence at age zero (p_d,normal_(a1=0)=0) . From this, the prevalence at age 1 is calculated from eq. 2, but using i_normal,d_(a1) instead of i_d_(a1). Next, the obtained prevalence at age 1 (p_d,normal_(a2)) can be used as p_d,normal_(a1) to obtain the prevalence at age 2, etc. This is continued until the prevalence in those with normal weight has been calculated for each age.


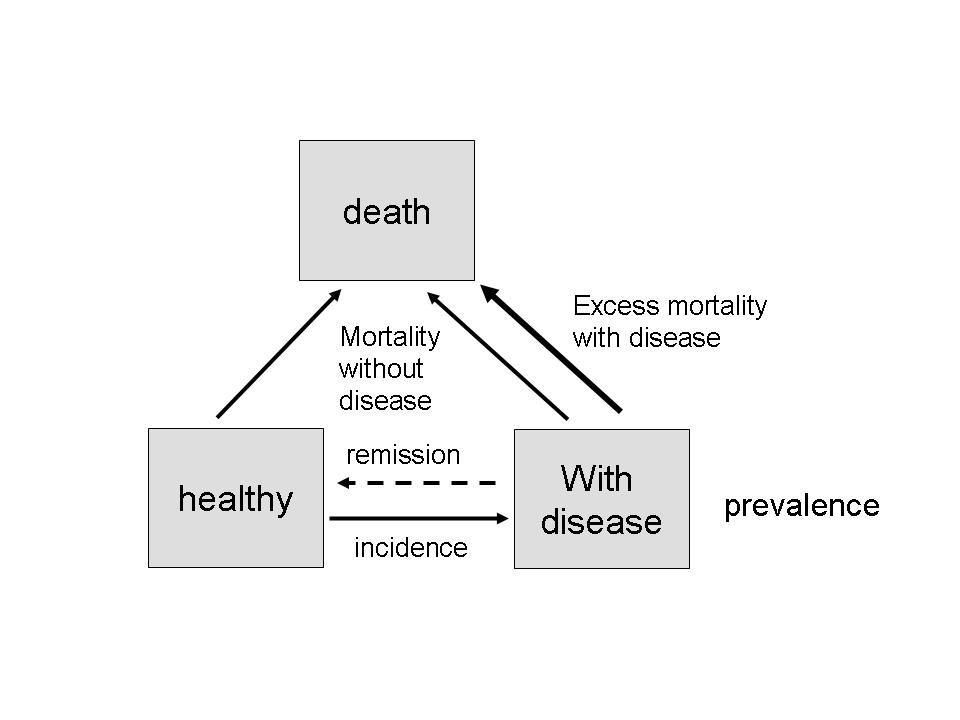


Fig. 2 Incidence-Prevalence-Mortality (IPM) model

**Determining costs of illness**

The OBCOST tool uses the top down approach for determining costs of illness. This approach assigns the costs on national level to single diseases, and determines which part of these diseases is due to overweight using the PAP. The proposed method takes age and gender into account; thereby the confounding effect of age is accounted for, as is the effect of diminishing relative risks with age. In principle, the method can be extended to take more confounding factors into account, but at the cost of needing detailed data that are not generally available.

The international comparability of health care costs is an important issue. Cross-country comparisons of costs of illness in general and costs of overweight in particular should be based on comparable definitions of health care and health expenditure. The use of a standardized health accounting framework is required. The OECD System of Health Accounts (SHA) [[3](#_ENREF_3)] is recommended for this purpose. However also within the SHA framework, it needs to be ensured that expenditures are indeed comparable. For instance, many variations exist in accounting the costs of nursing and residential care. At this moment, expenditure comparisons should therefore be restricted to curative care [[4](#_ENREF_4)]. Curative care expenditures need to be studied in detail as well to see whether, for example, one country includes paramedical care and another country does not.

**The OBCOST software**

The OBCOST tool consists of a Microsoft Excel file that uses macros to perform automatic calculations. The program consists of seven worksheets. Data has to be entered in the five datasheets (BMI and population data, relative risks, mortality data, disease data and cost data). When entering disease data, it should be indicated at the top whether it regards prevalence or incidence data. When entering cost data, it should be indicated at the top whether it regards total costs or costs per capita. When all data is entered, the sheet ‘main menu’ can be used to calculate total costs and costs of pre-obesity, obesity and overweight. In case more detailed information is necessary, the worksheet ‘detailed information’ provides more information about percentages and costs by disease. The program will issue a warning after pressing the calculate button in case of misuse of data, missing data or when prevalence data is not smooth enough. In case of missing data and no proper estimates are available, entering a zero will still enable the OBCOST software to function. This also provides the opportunity to exclude diseases from calculations.

**References**

1. Barendregt, J.J., Van Oortmarssen, G.J., Vos, T., Murray, C.J.: A generic model for the assessment of disease epidemiology: the computational basis of DisMod II. Popul Health Metr **1**(1), 4 (2003).

2. Hoogenveen R.T., Gijsen R., van Genugten M.L.L., Kommer G.J., Schouten J.S.A.G., de Hollander A.E.M.: Dutch DisMod. Constructing a set of consistent data for chronic disease modelling. In. RIVM, Bilthoven, (2000)

3. OECD, Eurostat, WHO: A System of Health Accounts. In. OECD Publishing (2011), doi: 10.1787/9789264116016-en,

4. Heijink, R., Noethen, M., Renaud, T., Koopmanschap, M., Polder, J.: Cost of illness: an international comparison. Australia, Canada, France, Germany and The Netherlands. Health Policy **88**(1), 49-61 (2008). doi:10.1016/j.healthpol.2008.02.012
