# Supplementary material for: Health care costs attributable to overweight calculated in a standardized way for three European countries
Source: Eur J Health Econ. 2014 Nov 29;17(1):61–9. doi: 10.1007/s10198-014-0655-8 (PMC4705131; doi:10.1007/s10198-014-0655-8)
Supplement: Supplementary file 2 — Supplementary material 2 (DOCX 37 kb) [file 10198_2014_655_MOESM2_ESM.docx]

**Online Resource 2**

**Relative risks entered into the OBCOST tool**

**Journal**

European Journal of Health Economics

# **Title**

# Health care costs attributable to overweight calculated in a standardized way for three European countries

# **Authors**

# M. Lette, W.J.E. Bemelmans, J. Breda, L.C.J. Slobbe, J. Dias, H.C. Boshuizen

#

# **Corresponding author**

# Manon Lette, MSc, National Institute for Public Health and the Environment, Centre for Nutrition, Prevention and Health Services, PO Box 1, 3720 BA Bilthoven, The Netherlands

e-mail: [manon.lette@rivm.nl](mailto:manon.lette@rivm.nl)

**Relative risks entered into OBCOST tool**

Data on relative risks is based both on data from the WHO and on data from the RIVM chronic disease modelling. These figures have been used as example relative risks in the OBCOST model. They can be replaced by values that the user might find more suitable. Where available, we added the figures taken from the WHO Comparative Quantification of Health Risks project [[3](#_ENREF_3)].

Ischemic Heart Disease

|  | BMI (kg/m^2^) | | | | | |
| --- | --- | --- | --- | --- | --- | --- |
|  | < 25 | | 25-30 | | ≥ 30 | |
| Age | Men | Women | Men | Women | Men | Women |
| **RIVM** |  |  |  |  |  |  |
| 0-34 | 1.00 | 1.00 | 1.41 | 1.48 | 2.12 | 2.31 |
| 35-39 | 1.00 | 1.00 | 1.41 | 1.49 | 2.25 | 2.47 |
| 40-44 | 1.00 | 1.00 | 1.38 | 1.46 | 2.15 | 2.43 |
| 45-49 | 1.00 | 1.00 | 1.34 | 1.40 | 2.04 | 2.19 |
| 50-54 | 1.00 | 1.00 | 1.31 | 1.35 | 1.87 | 2.05 |
| 55-59 | 1.00 | 1.00 | 1.26 | 1.29 | 1.67 | 1.83 |
| 60-64 | 1.00 | 1.00 | 1.19 | 1.22 | 1.54 | 1.61 |
| 65-69 | 1.00 | 1.00 | 1.17 | 1.19 | 1.38 | 1.46 |
| 70-74 | 1.00 | 1.00 | 1.13 | 1.15 | 1.30 | 1.35 |
| 75-79 | 1.00 | 1.00 | 1.09 | 1.10 | 1.21 | 1.24 |
| 80-84 | 1.00 | 1.00 | 1.06 | 1.07 | 1.13 | 1.14 |
| 85+ | 1.00 | 1.00 | 1.02 | 1.02 | 1.04 | 1.04 |
| **WHO** |  |  |  |  |  |  |
| <65^a^ | 1.00 | 1.00 | 1.35 | 1.40 | 1.80 | 2.00 |
| >65^a^ | 1.00 | 1.00 | 1.00 | 1.00 | 1.20 | 1.25 |
| All ages^b^ | 1.00 | 1.00 | - | - | 3.2 | 1.50 |

^a^ Based on Australian review

^b^ Based on UK review

**Stroke**

|  | BMI (kg/m^2^) | | | | | |
| --- | --- | --- | --- | --- | --- | --- |
| **RIVM** |  | |  | |  | |
|  | < 25 | | 25-30 | | ≥ 30 | |
| Age | Men | Women | Men | Women | Men | Women |
| 0-34 | 1.00 | 1.00 | 1.08 | 1.09 | 1.18 | 1.21 |
| 35-39 | 1.00 | 1.00 | 1.09 | 1.10 | 1.22 | 1.25 |
| 40-44 | 1.00 | 1.00 | 1.10 | 1.12 | 1.25 | 1.29 |
| 45-49 | 1.00 | 1.00 | 1.10 | 1.12 | 1.27 | 1.30 |
| 50-54 | 1.00 | 1.00 | 1.11 | 1.13 | 1.28 | 1.33 |
| 55-59 | 1.00 | 1.00 | 1.13 | 1.15 | 1.31 | 1.38 |
| 60-64 | 1.00 | 1.00 | 1.13 | 1.15 | 1.36 | 1.40 |
| 65-69 | 1.00 | 1.00 | 1.14 | 1.15 | 1.29 | 1.35 |
| 70-74 | 1.00 | 1.00 | 1.10 | 1.11 | 1.21 | 1.25 |
| 75-79 | 1.00 | 1.00 | 1.04 | 1.05 | 1.09 | 1.11 |
| 80-84 | 1.00 | 1.00 | 1.00 | 1.00 | 1.00 | 1.00 |
| 85+ | 1.00 | 1.00 | 1.00 | 1.00 | 1.00 | 1.00 |
| WHO |  |  |  |  |  |  |
| <65^a,b^ | 1.00 | 1.00 | 1.35 | 1.25 | 1.50 | 1.60 |
| >65^a,b^ | 1.00 | 1.00 | 1.00 | 1.00 | 1.15 | 1.20 |
| All ages^c^ | 1.00 | 1.00 | - | - | 1.3 | 1.3 |

^a^ Only ischemic stroke included

^b^ Based on Australian review

^c^ Based on UK review

**Hypertension**

|  | BMI (kg/m^2^) | | | | | |
| --- | --- | --- | --- | --- | --- | --- |
|  | < 25 | | 25-30 | | ≥ 30 | |
| Age | Men | Women | Men | Women | Men | Women |
| 0-34 | 1.00 | 1.00 | 1.22 | 1.25 | 1.61 | 1.61 |
| 35-39 | 1.00 | 1.00 | 1.21 | 1.25 | 1.57 | 1.65 |
| 40-44 | 1.00 | 1.00 | 1.20 | 1.24 | 1.54 | 1.65 |
| 45-49 | 1.00 | 1.00 | 1.19 | 1.22 | 1.53 | 1.60 |
| 50-54 | 1.00 | 1.00 | 1.20 | 1.22 | 1.52 | 1.62 |
| 55-59 | 1.00 | 1.00 | 1.20 | 1.22 | 1.49 | 1.59 |
| 60-64 | 1.00 | 1.00 | 1.18 | 1.20 | 1.48 | 1.55 |
| 65-69 | 1.00 | 1.00 | 1.20 | 1.22 | 1.43 | 1.53 |
| 70-74 | 1.00 | 1.00 | 1.20 | 1.22 | 1.46 | 1.56 |
| 75-79 | 1.00 | 1.00 | 1.20 | 1.22 | 1.48 | 1.56 |
| 80-84 | 1.00 | 1.00 | 1.23 | 1.25 | 1.54 | 1.57 |
| 85+ | 1.00 | 1.00 | 1.23 | 1.25 | 1.54 | 1.57 |
| **WHO** |  |  |  |  |  |  |
| <65^a^ | 1.00 | 1.00 | 1.40 | 1.40 | 2.35 | 2.35 |
| >65^a^ | 1.00 | 1.00 | 1.40 | 1.40 | 2.35 | 2.35 |
| All ages^b^ | 1.00 | 1.00 | - | - | 2.6 | 4.2 |

^a^ Based on Australian review

^b^ Based on UK review

**Colon Cancer**

| BMI (kg/m^2^) | | | | | | | | | |
| --- | --- | --- | --- | --- | --- | --- | --- | --- | --- |
|  | WHO | | | RIVM | | | | | |
|  | < 25 | 25-30 | ≥ 30 | < 25 | | 25-30 | | ≥ 30 | |
| Age |  |  |  | Men | Women | Men | Women | Men | Women |
| 0-34 | 1.00 | 1.15 | 1.33 | 1.00 | 1.00 | 1.22 | 1.25 | 1.53 | 1.61 |
| 35-39 | 1.00 | 1.15 | 1.33 | 1.00 | 1.00 | 1.21 | 1.25 | 1.57 | 1.65 |
| 40-44 | 1.00 | 1.15 | 1.33 | 1.00 | 1.00 | 1.20 | 1.24 | 1.54 | 1.65 |
| 45-49 | 1.00 | 1.15 | 1.33 | 1.00 | 1.00 | 1.19 | 1.22 | 1.53 | 1.60 |
| 50-54 | 1.00 | 1.15 | 1.33 | 1.00 | 1.00 | 1.20 | 1.22 | 1.52 | 1.62 |
| 55-59 | 1.00 | 1.15 | 1.33 | 1.00 | 1.00 | 1.20 | 1.22 | 1.49 | 1.59 |
| 60-64 | 1.00 | 1.15 | 1.33 | 1.00 | 1.00 | 1.22 | 1.25 | 1.53 | 1.61 |
| 65-69 | 1.00 | 1.15 | 1.33 | 1.00 | 1.00 | 1.20 | 1.22 | 1.43 | 1.53 |
| 70-74 | 1.00 | 1.15 | 1.33 | 1.00 | 1.00 | 1.20 | 1.22 | 1.46 | 1.56 |
| 75-79 | 1.00 | 1.15 | 1.33 | 1.00 | 1.00 | 1.20 | 1.22 | 1.48 | 1.56 |
| 80-84 | 1.00 | 1.15 | 1.33 | 1.00 | 1.00 | 1.23 | 1.25 | 1.54 | 1.57 |
| 85+ | 1.00 | 1.15 | 1.33 | 1.00 | 1.00 | 1.23 | 1.25 | 1.54 | 1.57 |

**Breast cancer (women)**

**WHO data are for postmenopausal breast cancer**

|  | BMI (kg/m^2^) | | | | | |
| --- | --- | --- | --- | --- | --- | --- |
|  | WHO | | | RIVM | | |
| Age | < 25 | 25-30 | ≥ 30 | < 25 | 25-30 | ≥ 30 |
| 0-34 | 1.00 | 1.12 | 1.25 | 1.00 | 1.00 | 1.00 |
| 35-39 | 1.00 | 1.12 | 1.25 | 1.00 | 1.05 | 1.12 |
| 40-44 | 1.00 | 1.12 | 1.25 | 1.00 | 1.10 | 1.25 |
| 45-49 | 1.00 | 1.12 | 1.25 | 1.00 | 1.20 | 1.52 |
| 50-54 | 1.00 | 1.12 | 1.25 | 1.00 | 1.25 | 1.70 |
| 55-59 | 1.00 | 1.12 | 1.25 | 1.00 | 1.25 | 1.67 |
| 60-64 | 1.00 | 1.12 | 1.25 | 1.00 | 1.22 | 1.63 |
| 65-69 | 1.00 | 1.12 | 1.25 | 1.00 | 1.19 | 1.46 |
| 70-74 | 1.00 | 1.12 | 1.25 | 1.00 | 1.10 | 1.22 |
| 75-79 | 1.00 | 1.12 | 1.25 | 1.00 | 1.00 | 1.00 |
| 80-84 | 1.00 | 1.12 | 1.25 | 1.00 | 1.00 | 1.00 |
| 85+ | 1.00 | 1.12 | 1.25 | 1.00 | 1.00 | 1.00 |

**Endometrial cancer (women)**

|  | BMI (kg/m^2^) | | | | | |
| --- | --- | --- | --- | --- | --- | --- |
|  | WHO | | | RIVM^a^ | | |
| Age | < 25 | 25-30 | ≥ 30 | < 25 | 25-30 | ≥ 30 |
| All | 1.00 | 1.59 | 2.52 | 1.00 | - | - |

^a^ In the OBCOST tool the WHO data was used.

**Kidney Cancer**

| BMI (kg/m^2^) | | | | | | | | | |
| --- | --- | --- | --- | --- | --- | --- | --- | --- | --- |
|  | WHO | | | RIVM | | | | | |
|  | < 25 | 25-30 | ≥ 30 | < 25 | | 25-30 | | ≥ 30 | |
| Age |  |  |  | Men | Women | Men | Women | Men | Women |
| 0-34 | 1.00 | 1.36 | 1.84 | 1.00 | 1.00 | 1.79 | 1.79 | 3.57 | 3.57 |
| 35-39 | 1.00 | 1.36 | 1.84 | 1.00 | 1.00 | 1.57 | 1.57 | 2.90 | 2.90 |
| 40-44 | 1.00 | 1.36 | 1.84 | 1.00 | 1.00 | 1.43 | 1.43 | 2.32 | 2.32 |
| 45-49 | 1.00 | 1.36 | 1.84 | 1.00 | 1.00 | 1.31 | 1.31 | 1.93 | 1.93 |
| 50-54 | 1.00 | 1.36 | 1.84 | 1.00 | 1.00 | 1.27 | 1.27 | 1.74 | 1.74 |
| 55-59 | 1.00 | 1.36 | 1.84 | 1.00 | 1.00 | 1.17 | 1.17 | 1.42 | 1.42 |
| 60-64 | 1.00 | 1.36 | 1.84 | 1.00 | 1.00 | 1.12 | 1.12 | 1.30 | 1.30 |
| 65-69 | 1.00 | 1.36 | 1.84 | 1.00 | 1.00 | 1.08 | 1.08 | 1.18 | 1.18 |
| 70-74 | 1.00 | 1.36 | 1.84 | 1.00 | 1.00 | 1.04 | 1.04 | 1.09 | 1.09 |
| 75-79 | 1.00 | 1.36 | 1.84 | 1.00 | 1.00 | 1.00 | 1.00 | 1.00 | 1.00 |
| 80-84 | 1.00 | 1.36 | 1.84 | 1.00 | 1.00 | 1.00 | 1.00 | 1.00 | 1.00 |
| 85+ | 1.00 | 1.36 | 1.84 | 1.00 | 1.00 | 1.00 | 1.00 | 1.00 | 1.00 |

**Diabetes**

|  | BMI (kg/m^2^) | | | | | |
| --- | --- | --- | --- | --- | --- | --- |
|  | < 25 | | 25-30 | | ≥ 30 | |
| Age | Men | Women | Men | Women | Men | Women |
| **RIVM** |  |  |  |  |  |  |
| 0-19 | 1.00 | 1.00 | 1.00 | 1.00 | 1.00 | 1.00 |
| 20-24 | 1.00 | 1.00 | 4.94 | 3.73 | 21.13 | 23.69 |
| 25-29 | 1.00 | 1.00 | 3.65 | 3.70 | 17.43 | 19.33 |
| 30-34 | 1.00 | 1.00 | 3.57 | 3.33 | 16.18 | 13.26 |
| 35-39 | 1.00 | 1.00 | 3.18 | 3.15 | 15.57 | 13.76 |
| 40-44 | 1.00 | 1.00 | 2.84 | 2.94 | 11.79 | 12.33 |
| 45-49 | 1.00 | 1.00 | 2.57 | 2.63 | 9.74 | 9.53 |
| 50-54 | 1.00 | 1.00 | 2.43 | 2.49 | 7.84 | 8.85 |
| 55-59 | 1.00 | 1.00 | 2.24 | 2.30 | 5.91 | 6.97 |
| 60-64 | 1.00 | 1.00 | 1.88 | 1.98 | 4.61 | 5.17 |
| 65-69 | 1.00 | 1.00 | 1.75 | 1.85 | 3.11 | 3.81 |
| 70-74 | 1.00 | 1.00 | 1.56 | 1.63 | 2.52 | 2.93 |
| 75-79 | 1.00 | 1.00 | 1.37 | 1.41 | 1.95 | 2.13 |
| 80-84 | 1.00 | 1.00 | 1.23 | 1.24 | 1.53 | 1.56 |
| 85+ | 1.00 | 1.00 | 1.06 | 1.06 | 1.12 | 1.13 |
| **WHO**^a^ |  |  |  |  |  |  |
| <65^b^ | 1.00 | 1.00 | 1.8 | 1.8 | 3.2 | 3.2 |
| >65^b^ | 1.00 | 1.00 | 1.8 | 1.8 | 3.2 | 3.2 |
| All ages^c^ | 1.00 | 1.00 | - | - | 5.2 | 5.2 |

^a^ Relative risk of incidence of diabetes type II

^b^ Based on Australian review

^c^ Based on UK review

**Osteoarthritis**

|  | BMI (kg/m^2^) | | | | | |
| --- | --- | --- | --- | --- | --- | --- |
|  | < 25 | | 25-30 | | ≥ 30 | |
| Age | Men | Women | Men | Women | Men | Women |
| 0-34 | 1.00 | 1.00 | 1.91 | 4.54 | 1.93 | 4.59 |
| 35-39 | 1.00 | 1.00 | 1.76 | 1.79 | 4.21 | 4.31 |
| 40-44 | 1.00 | 1.00 | 1.63 | 1.66 | 3.40 | 3.51 |
| 45-49 | 1.00 | 1.00 | 1.53 | 1.55 | 2.94 | 3.02 |
| 50-54 | 1.00 | 1.00 | 1.50 | 1.53 | 2.66 | 2.77 |
| 55-59 | 1.00 | 1.00 | 1.43 | 1.45 | 2.23 | 2.33 |
| 60-64 | 1.00 | 1.00 | 1.35 | 1.37 | 2.10 | 2.18 |
| 65-69 | 1.00 | 1.00 | 1.33 | 1.35 | 1.78 | 1.83 |
| 70-74 | 1.00 | 1.00 | 1.30 | 1.32 | 1.75 | 1.79 |
| 75-79 | 1.00 | 1.00 | 1.25 | 1.26 | 1.61 | 1.63 |
| 80-84 | 1.00 | 1.00 | 1.23 | 1.23 | 1.54 | 1.52 |
| 85+ | 1.00 | 1.00 | 1.19 | 1.15 | 1.43 | 1.34 |
