# Supplementary material for: Health care costs attributable to overweight calculated in a standardized way for three European countries
Source: Eur J Health Econ. 2014 Nov 29;17(1):61–9. doi: 10.1007/s10198-014-0655-8 (PMC4705131; doi:10.1007/s10198-014-0655-8)
Supplement: Supplementary file 3 — Supplementary material 3 (DOCX 23 kb) [file 10198_2014_655_MOESM3_ESM.docx]

**Online Resource 3**

**Justification of input data**

**Journal**

European Journal of Health Economics

# **Title**

# Health care costs attributable to overweight calculated in a standardized way for three European countries

# **Authors**

# M. Lette, W.J.E. Bemelmans, J. Breda, L.C.J. Slobbe, J. Dias, H.C. Boshuizen

#

# **Corresponding author**

# Manon Lette, MSc, National Institute for Public Health and the Environment, Centre for Nutrition, Prevention and Health Services, PO Box 1, 3720 BA Bilthoven, The Netherlands

# e-mail: [manon.lette@rivm.nl](mailto:manon.lette@rivm.nl)

**Input data**

For each country, the following data were provided: population data, BMI prevalence data, mortality data of included diseases, prevalence/incidence data of included diseases and cost data of included diseases. Data were provided by the following sources:

The Netherlands: Population data, BMI prevalence data and mortality data were retrieved from Statistics Netherlands (Centraal Bureau voor Statistiek) [[1](#_ENREF_1)]. Prevalence data for all diseases except the cancers were based on five general practitioners registrations [[2](#_ENREF_2)]. Incidence data for the cancers were retrieved from the Dutch National Cancer Registry of Integraal Kankercentrum Nederland [[3](#_ENREF_3)]. Cost data were retrieved from the Dutch interactive cost-of-illness (kosten van ziekten) website [[4](#_ENREF_4)].

Germany: population data, BMI prevalence data, mortality data for all diseases except cancers, disease data for hypertension and osteoarthritis and cost data were retrieved from the German Statistical Office (Gesundheitsberichterstattung des Bundes) [[5](#_ENREF_5)]. Mortality data for all four cancers and diseases data for endometrial cancer and kidney cancer were retrieved from the German National Cancer Registry of the Robert Koch Institut [[6](#_ENREF_6)]. The rest of the disease data were retrieved from the database used for the Dynamic Modelling for Health Impact Assessment (Dynamo-HIA) project [[7](#_ENREF_7)].

Czech Republic: Population data and mortality data were retrieved from the Czech Statistical Office [[8](#_ENREF_8)]. BMI prevalence data and disease data for all diseases except cancers were retrieved form EHIS surveys [[9](#_ENREF_9)]. Disease data for the four cancers were retrieved from the Czech National Cancer Registry [[10](#_ENREF_10)]. Cost data were provided by an unpublished computation based on the data from the System of Health Accounts [[11](#_ENREF_11)].

**Methods of estimating missing data**

In order for the OBCOST tool to function, it is required that all entry fields are filled with data. Often, provided data was incomplete and estimations were made in order to fill all the required entry fields. The following methods were used:

1. Filling empty age categories for population data

Sometimes population numbers were reported with the eldest ages as an aggregate group (for example the aggregate group 90+ instead of the original age groups 90-94, 95-99 and 100+). The number provided in the aggregate age group was divided over the original age groups according to the proportions provided by the WHO world standard population distribution [[12](#_ENREF_12)].

1. Filling empty age categories from aggregate age groups for prevalence/incidence and mortality data

Prevalence/incidence data and mortality data were sometimes reported in aggregate age groups instead of the original age groups as required in the OBCOST tool. In order to fill all required age groups, it was assumed that the rate provided for the aggregate age group could be applied to all original age groups within this aggregate age group. If for example rate *x* was provided for age group 15-25, this rate was applied to both age group 15-19 and age group 20-24.

1. Filling missing data for younger age groups

Sometimes, BMI prevalence data, mortality data of included diseases or prevalence/incidence data of included diseases for the younger age groups (0-15) was missing. In order to fill the required entry cells, the rate provided in the first successive age category was applied to the previous age categories as well. For example, if data was missing for the age groups 0-4, 5-9 and 10-14, rate *z* provided for age category 15-19 was applied to the previous age categories.

1. Filling empty age categories from aggregate age groups for cost data

When costs data was provided in aggregated age groups, the number for this aggregate age group was divided by the number of original age groups within this aggregate age group. This result was applied to all original age groups within the aggregate age group. For example, if number *y* was provided for age group 15-30, then *y*/3 was applied to age groups 15-19, 20-24 and 25-29.

1. Smoothing prevalence data

OBCOST uses the method of back-calculation of incidence data from prevalence data. This is only possible if the prevalence data are sufficiently smooth. The prevalence should only decrease with age if the excess mortality from the disease is large enough to make the prevalence rate decrease with age. This is usually only the case at the oldest ages. For all other ages it is recommended to enter only monotonously increasing prevalence rates. If necessary, smoothing methods are to be used on the data first. In the data provided, some slight deviations were found. In order to smooth this data, a quick and dirty fix was used by averaging the prevalence rate causing error with the one in the previous age group. If for example rate *a* was provided for age group 55-59 and the lower rate *b* was provided for age group 60-64, the average of *a* and *b* was applied to both these age groups.

**References**

1. CBS: Figures by Theme. <http://www.cbs.nl/en-GB/menu/cijfers/cijfers-per-thema/default.htm> (2012).

2. RIVM: Volksgezondheid Toekomst Verkenning, Nationaal Kompas Volksgezondheid. <http://www.nationaalkompas.nl> (2012). 2012

3. IKNL: Cijfers over Kanker. <http://cijfersoverkanker.nl/> (2011). 2012

4. RIVM: Kosten van Ziekten. <http://www.kostenvanziekten.nl> (2007). 2012

5. GBE-Bund: The Information System of the Federal Health Monitoring. <http://www.gbe-bund.de/gbe10/pkg_isgbe5.prc_isgbe?p_uid=gast&p_aid=4711&p_sprache=E>. 2012

6. RKI: Zentrum für Krebsregisterdaten. <http://www.krebsdaten.de/Krebs/EN/Home/homepage_node.html>. 2012

7. Dynamo-HIA: Dynamo-HIA software. <http://www.dynamo-hia.eu/object_class/dyhia_software.html>. 2012

8. CZSO: Public Database. <http://vdb.czso.cz/vdbvo/en/uvod.jsp>. 2012

9. Eurostat: Statistics. <http://epp.eurostat.ec.europa.eu/portal/page/portal/statistics/search_database>. 2012

10. Dusek, L., Muzík, J., Kubásek, M., Koptíková, J., Zaloedík, J., Vyzula, R.: Epidemiology of Malignant Tumours in the Czech Republic [online]. Masaryk University, Czech Republic. <http://www.svod.cz> (2005). 2012

11. Roubal, T.: Own computation based on the Czech Statistical Office System of Health Accounts, Czech Republic, Data 2010, unpublished. In.

12. Ahmad, O.B., Boschi-Pinto, C., Lopez, A.D., Murray, C.J.L., Lozano, R., Inoue, M.: Age standardization of rates: a new WHO standard. In. Global Programme on Evidence for Health Policy Discussion Paper Series: No. 31. World Health Organization, (2001)
